# Integrating accompanying patients into clinical oncology teams: limiting and facilitating factors

**DOI:** 10.1186/s12913-024-10624-w

**Published:** 2024-01-30

**Authors:** Marie-Pascale Pomey, Jesseca Paquette, Monica Iliescu Nelea, Cécile Vialaron, Rim Mourad, Karine Bouchard, Louise Normandin, Marie-Andrée Côté, Mado Desforges, Pénélope Pomey-Carpentier, Israël Fortin, Isabelle Ganache, Catherine Régis, Zeev Rosberger, Danielle Charpentier, Marie-France Vachon, Lynda Bélanger, Michel Dorval, Djahanchah P. Ghadiri, Mélanie Lavoie-Tremblay, Antoine Boivin, Jean-François Pelletier, Nicolas Fernandez, Alain M. Danino, Michèle de Guise

**Affiliations:** 1 Research Centre of the University of Montreal Hospital Centre, Montréal, QC Canada; 2 Centre d’excellence Sur Le Partenariat Avec Les Patients Et Le Public, Montréal, QC Canada; 3 Department of Health Policy, Management and Evaluation, School of Public Health, University of Montréal, Montréal, QC, CA Canada; 4 Centre Hospitalier Universitaire- CHU de Québec-Université Laval, Québec, QC Canada; 5 Centre Intégré, Universitaire de Santé Et Services Sociaux de L’Est-de-L’Île-de Montréal, Hôpital de Maisonneuve-Rosemont, Montréal, QC Canada; 6 Institut National d’excellence en Santé Et Services Sociaux (INESSS), Montréal, QC Canada; 7 Université de Montréal – Faculté de droit, Montréal, QC Canada; 8 Gerald Bronfman Department of Oncology, Lady Davis Institute for Medical Research, Jewish General Hospital &, McGill University, Montréal, QC Canada; 9 Centre Hospitalier Universitaire de Montréal (CHUM), Montréal, QC Canada; 10 Faculté de Pharmacie, Université Laval, Québec, QC Canada; 11 Centre de Recherche du CHU de Québec-Université Laval, Québec, QC Canada; 12 Centre de Recherche du CISSS Chaudière Appalaches, Lévis, QC Canada; 13 Department of Management, HEC Montréal, Montréal, QC Canada; 14 Faculté Des Sciences Infirmières, Université de Montréal, Montréal, QC Canada; 15 Institut Universitaire en Santé Mentale de Montréal, Montréal, QC Canada; 16 Department of Family and Emergency Medicine, Faculté de Médecine, Université de Montréal, Montréal, QC Canada; 17 Centre Intégré de Santé Et de Services Sociaux de La Montérégie-Ouest, St-Hubert, QC Canada; 18 Yale Program for Recovery & Community Health, New Haven, CT USA

**Keywords:** Accompanying patients, Patient advisor, Peer support, Oncology, Patient care experience, Clinical team, Facilitating factors, Barriers

## Abstract

**Objectives:**

Since 2018, four establishments in Quebec have been instrumental in implementing the PAROLE-Onco program, which introduced accompanying patients (APs) into healthcare teams to improve cancer patients’ experience. APs are patient advisors who have acquired specific experiential knowledge related to living with cancer, using services, and interacting with healthcare professionals. They are therefore in a unique and reliable position to be able to provide emotional, informational, cognitive and navigational support to patients who are dealing with cancer. We aimed to explore APs’ perspectives regarding the limiting and facilitating factors in terms of how they are integrated into the clinical oncology teams.

**Methods:**

A qualitative study based on semi-structured interviews and focus groups was conducted with 20 APs at the beginning of their intervention (T1) and, two years later, during a second data collection (T2). Limiting and facilitating factors of APs’ integration into clinical teams were analyzed in terms of governance, culture, resources and tools.

**Results:**

The limited factors raised by APs to be integrated into clinical teams include the following: confusion about the specific roles played by APs, lifting the egos of certain professionals who feel they are already doing what APs typically do, lack of identification of patient needs, absence of APs in project governance organizational boundaries, and team members' availability. Various communication challenges were also raised, resulting in the program being inadequately promoted among patients. Also mentioned as limiting factors were the lack of time, space and compensation. Creating opportunities for team members to meet with APs, building trust and teaching team members how APs’ activities complement theirs were enhancing factors. Other facilitators include APs being involved in decision-making committees, being leaders in promoting the PAROLE-Onco program to patients and clinical team members and creating opportunities to communicate with team members to help enhance their work and provide feedback to improve patient services. Awareness of APs’ added value for the team and patients is also a key facilitator. Regarding tools, offering accompanying services by telephone allows both patients and APs to benefit from the flexibility they need.

**Conclusion:**

Over time, APs were able to identify optimal factors for successful implementation. Recommendations include APs and professionals working in co-construction on organization, leadership, resources and status factors. This could help catalyze a change in culture within health establishments and allow people dealing with cancer to benefit from the experiential knowledge of other patients within their clinical team.

**Supplementary Information:**

The online version contains supplementary material available at 10.1186/s12913-024-10624-w.

Contributions to the literature

- By being among the most significantly impacted by the implementation of the PAROLE-Onco program, APs are in a strong position to evaluate the program’s implementation and identify the factors that would best facilitate their integration.
- Misunderstandings about APs’ roles can delay a change in culture in healthcare establishments and make APs’ accompanying services more challenging to promote.
- Assigning certain powers to APs, via co-construction and co-decision methods, is conducive to ensuring a successful change in culture within healthcare establishments.
- Working proactively with APs on organization, leadership, resources, and status factors will allow patients dealing with cancer to benefit from the experiential knowledge of other patients within their clinical team.

## Introduction

It is estimated that in 2022, 60,000 Quebecers were diagnosed with cancer, which represents 158 new cases per day [1]. This number has been on the rise for several years and is expected to increase even further in the coming years due to testing delays following the pandemic [2]. The assessment of cancer patients’ experience highlighted that the most lacking aspect among the six areas of patient experience assessed in health and social service organizations in Quebec and across Canada was that of emotional support [3]. This need is all the more significant in the context of a pandemic in which patients expect and hope to receive emotional support.

To meet this need, the PAROLE-Onco program [4] was developed as part of a project funded by the Canadian Institutes of Health Research and the provincial Ministry of Health and Social Services and set up in four different healthcare establishments in Quebec, Canada. It revolves around integrating into clinical teams accompanying patients (APs), who are patients having acquired specific experiential knowledge related to living with cancer, using services, and interacting with healthcare professionals (HPs) [5]. They are trained to be active listeners, to know when and how to tell their story, to help patients navigate their care, and to liaise with the clinical team. In oncology, peer support has usually been provided by "patient navigators," a role typically assigned to nurses, social workers, educators, as well as to former patients [6]. In contrast, APs are patients themselves who are in a position to transform their experience into a resource available to other people, which contributes not only to enhancing their own health and self-esteem but also the health of patients undergoing a cancer treatment journey. In the program, selected APs were trained and coached on how to intervene with patients. At the same time, they were given space to innovate in their own ways to accompany patients based on their specific experiential knowledge. Since 2019, HPs have introduced, during medical appointments with patients, AP accompanying services as an additional resource, and patients are absolutely free to accept or refuse such a resource. Research coordinators or clinical staff members collected essential clinical data on patients who had anonymously and confidentially consented to participate to match them with an AP with a similar profile. Patients then made appointments with their AP according to their needs. Initially, each site recruited at least two APs and, over time, recruited and trained additional APs to meet patients’ needs.

By helping patients access healthcare, patient navigators have facilitated and hence accelerated diagnosis and treatment journeys [7]. Indeed, patients have benefited from these programs as it has been reported that such programs have been instrumental in increasing adherence to treatment [8], bringing comfort [9] and guiding patients through the healthcare system [7]. APs can also improve patients’ quality of life by promoting healthy lifestyle habits and reducing symptoms of anxiety and depression [10]. Our parallel study on APs’ perspectives regarding their activities within the clinical oncology teams has shown that APs provide emotional, informational, cognitive and navigational support to patients by mobilizing their experiential knowledge [11]. APs help patients feel understood and supported. They alleviate stress and become partners in their care. They also alleviate the clinical team’s workload by providing a complementary service through emotional support, which can calm patients down and help them be better prepared for their appointments with health professionals (HPs). APs communicate additional information about their patients’ health journey, which makes appointments more efficient for HPs. When APs accompany patients, they feel as if they can make a difference in patients’ lives. Their activities are often perceived as an opportunity to give back but also as an excellent way of giving meaning to their own experience, thereby serving as a learning experience.

The positive effects of APs’ intervention on patients, on the clinical team and on themselves, has resulted in mixed reactions by APs themselves. While a few studies have assessed the barriers and facilitating factors of integrating peers in different healthcare settings [12, 13], including oncology [14–16], APs’ perspectives on oncology clinical teams continue to be poorly documented. This study addresses the inner settings, individuals, and implementation process domains and the related constructs regarding implementation of the PAROLE-Onco program in four institutions, in light of the fact that the literature on APs’ perspectives regarding limiting and facilitating factors in their integration into the clinical oncology teams is insufficient.

### Theoretical frameworks

We used the framework developed by Pomey et al. [17] based on Parsons’ model [18] to interpret our results. This framework identified four categories of factors relevant to analyzing the implementation of interventions: (1) governance, defined as “the conduct of collective action from a position of authority” [19] (2) culture, defined as “underlying beliefs, values, norms and behaviours” including physicians’ involvement [20], (3) resources, defined as human, financial, infrastructural or informational, and (4) tools, defined as the instruments or procedures considered helpful in implementing a strategy. In addition, we mobilized the Consolidated Framework for Implementation Research (CFIR) [21, 22] to discuss our results to be sure to cover all aspects of factors likely to interact during the integration of complex intervention.

## Methods

Data was collected on two separate occasions, when the PAROLE-Onco project was first implemented (T1), and two years later (T2). Our approach to reporting this qualitative study is based on the Standards for Reporting Qualitative Research (SRQR; Additional file 1 [23].

### Settings

The four establishments included in this study are: the Centre hospitalier de l’Université de Montréal (E1), the Centre Hospitalier Universitaire de Québec-Université Laval (E2), the Centre intégré universitaire de santé et de services sociaux (CIUSSS) de l’Est-de-l’Île-de-Montréal (E3), and the CIUSSS de la Mauricie-et-du-Centre-du-Québec (E4). Each establishment recruited their own APs (29 in total). The programs in which APs were involved include two in breast cancer (E1 and E4), one in breast oncogenetics (E2), and one in breast and gynecological cancers (E3).

During T1, E1 had 5 APs in the breast cancer program, E2 had 4 APs in the breast oncogenetics program, E3 had 5 APs in the breast and gynecological cancer program, and E4 had 3 APs in the breast cancer program again. During T2, E1 had 9 APs in the breast cancer program, E2 had 2 APs in the breast oncogenetics program, E3 had 14 APs in the breast and gynecological cancer program, and T4 had 1 AP in the breast cancer program.

### Data collection

The study employed semi-structured interviews and focus group discussions to collect data, as described in our previous paper [11]. APs from the four establishments were invited to participate in both T1 and T2. Participants were contacted by email or phone and provided an electronically signed consent form, which was approved by the Research Ethics Committee (17.260). No compensation was offered for participation. All participants gave their consent to take part in the research and be recorded. Due to the COVID-19 pandemic, interviews were conducted by telephone or videoconference, and focus group discussions were carried out through videoconference. During T1, the questions (found in the supplementary material) were designed to identify APs’ perceptions of the limiting and facilitating factors of their integration into the clinical oncology teams. The questions were co-created and pilot-tested with two patient-researchers who were included in the research team. The T1 data collection events took place four months after APs were introduced into the four establishments. Two years later, during T2, the data collection aimed, by presenting the T1 results, to evaluate changes in APs’ perspectives on the limiting and facilitating factors of their integration into the clinical oncology teams. APs discussed changes in elements since the new APs joined the team or the emergence of new elements. Thus, no interview guide was used in T2. The interview transcripts and focus group discussions were prepared, and all data collection events were conducted in French before being translated into English.

### Data analysis

To analyze data, we followed the six-step guideline of Braun and Clarke [24]. First, all interviews were transcribed to familiarize us with the data. Second, several meetings between the authors, including two patient-researchers, were held to construct the codebook that contained two main categories: (1) the limiting and (2) the facilitating factors of their integration into the clinical oncology teams. We then used a thematic analysis approach to better “understand a set of experiences, thoughts, or behaviors” pertaining to these categories [25]. We used an inductive approach to theme identification — or patterned responses that occurred in the dataset [25]. Coding was done using the QDA Miner Software (version 6.0.2.). Steps four and five consisted of grouping some themes together according to the framework developed by Pomey et al. [17].

## Results

### General results

In total, for the two rounds of data collection (T1/T2), we were able to interview 20 different APs (T1: *n* = 10, T2: *n* = 10).

There were two types of data collection events: focus groups and interviews. In T1 (the first round of data collection), there were 2 focus groups with a total of 7 participations, and 8 interviews with a total of 8 participations. This means that there was a total of 15 participations in T1. In T2 (the second round of data collection), there were 5 focus groups with a total of 19 participations, but no interviews. This means that there was a total of 19 participations in T2.

It was found that out of the total of 15 participations in T1, 5 APs participated in 2 events. It can thus be concluded that there were only 10 different APs in T1. Similarly, in T2, out of the total of 19 participations, 3 APs participated in 2 events, indicating that there were 16 different APs in T2. Further analysis revealed that 6 of these APs also participated in T1. Hence, it can be inferred that there were 10 new APs that were interviewed in T2 since T1. In phase T1 of the study, all 10 APs who were involved in the 4 healthcare establishments and had accompanied patients agreed to participate. One focus group with E4 was conducted in June 2019 (*n* = 3 participants). Another focus group was held in September 2019 with E1, E2, and E3 (*n* = 4). The 2 focus groups were facilitated by the lead researcher or a research assistant and lasted 58 and 178 min, respectively. Additionally, 8 individual interviews were conducted between April and May 2020, ranging from 30 to 63 min in duration. Out of the 10 APs, 5 participated in both the individual interviews and focus group data collection events.

During T2, 16 out of 20 APs who accompanied patients agreed to participate in the study. Four did not respond to our invitation. Out of the 16 participants, 6 had taken part in T1. The remaining 4 APs who had participated in T1 were not invited to participate in T2 due to personal reasons, as they were no longer involved in the PAROLE-Onco project. Therefore, 10 new APs were interviewed in T2. An initial focus group was conducted in September 2021 with E1 and E3, which had 3 participants and lasted for 35 min. At the time of the focus group, the APs had been accompanying patients for a period ranging from 12 to 22 months. Additionally, 4 other focus groups were held between March and May 2022 for each establishment, involving a total of 16 participants and lasting between 80 and 115 min. The APs had been involved for a range of 6 to 32 months during this period. The focus groups were led by either the principal researcher or a research assistant. Three out of the 16 APs participated in 2 data-collection events.

The data shows that the number of participants increased in all establishments except for E4, where the number of participants decreased from 3 in T1 to 1 in T2. In E1, the number of participants increased from 4 in T1 to 7 in T2. In E2, the number of participants increased from 1 in T1 to 2 in T2. In E3, the number of participants increased from 2 in T1 to 6 in T2. Therefore, the largest increase in the number of participants was observed in E3, which saw an increase of 4 participants from T1 to T2.

The majority of participants in both rounds of data collection were aged 55–64 years old (*n* = 5 in T1 and *n* = 7 in T2), followed by those aged 65–74 years old (*n* = 3 in T1 and *n* = 4 in T2). The number of participants born in the province of Quebec increased from 9 in T1 to 14 in T2. In terms of education, most participants in both T1 and T2 had a university degree. The number of participants who reported working full-time increased from 0 in T1 to 3 in T2, while the number of retired participants decreased from 7 in T1 to 6 in T2. Breast cancer was the most common cancer type among participants in both rounds of data collection (Table 1).

**Table 1:** Characteristics of the establishments

Establishment	Program	Number of APs in T1	Number of APs in T2
E1	Breast cancer	5	9
E2	Breast oncogenetics	4	2
E3	Breast and gynecological cancer	5	14
E4	Breast cancer	3	1

### Governance

#### Pushing boundaries

In both T1 and T2, APs mentioned feeling powerless inside a system that seemed too big for them, and that it was excessively difficult to take actions that could generate significant changes due to the administrative burden. Hence, APs considered that the organizational boundaries limited their role by preventing a certain cohesion between the work done by APs and that performed by the healthcare team, in addition to slowing down the decision-making process which is so critical to the project’s growth.

"There are maybe 50 people, and when I get to these organizational committees, I feel like I'm there because up there, they want patient-partners everywhere, but basically, if they could have none, that would be fine." (E3-03)

"When I sit on committees, it's a bit hard to get my bearings, to know who's who, how decisions are made and so on." (E1-01)

"We're not really involved in the organization, so our effect is minimal. The day we're really integrated, we'll be able to talk about success, but not now." (E4-03)

Some HPs also tried to integrate APs in decision-making committees, as evidenced by one AP who expressed that "*no decision should be made if there are no patient-partners*" (E3-02-T1), reflecting the sentiment of several other APs. However, to facilitate this integration, in T1, APs suggested that this integration be done slowly, because they "*don't want to hurt people and […] then have to go back twenty steps*" (E1-02-T1). In T2, APs reinforced the benefits of having joined in gradually as they could clearly define their role within the rest of the health team and reassure them that their patient-caregiver relationship would not be jeopardized by the APs’ involvement.

‘’Over time, the professionals began to understand why we needed to be present on committees and involved in the decision-making. We gradually took our place, without pushing anyone too hard, but by calmly bringing our ideas to the table and recognizing how our perspective could help them in implementing the intervention.’’ (E1-01-T2)

#### Shared leadership

Some APs also mentioned in T1 that there was a lack of energy put into the project, especially in integrating them into the team. It was, among others, the HPs’ lack of interest that explained why things were advancing slowly at the beginning.

‘’I took part in a lot of meetings at the beginning, but I had the impression that they weren't really helping the project to move forward. During the meetings, I gave my point of view, but I didn't see any action to implement it.’’ (E3-02-T1)

‘’The slowness of the project's implementation is, I think, attributable to the clinical staff’s lack of involvement, which partially explained the few referrals made to the APs.’’ (E4-02-T1)

One facilitating factor suggested in T1 for project implementation is that the clinical team, not the managers, must prompt the initiative mentioned by APs that they should be involved in promoting the project, and that it all starts by gaining the team members’ trust: “*The pivot nurse asked me to accompany her on her consultations. She would see the women and then suggest that they meet with me."* (E4-03-T1).

In T2, they specified a need for the physical presence of an AP coordinator acting as a carrier of the project and a leader in the hope that a certain degree of motivation towards its continuous betterment would be maintained and reinforced.

‘’A few months ago, the facility hired a coordinator to facilitate our contact with patients. This individual promotes the program to clinicians and gives us access to clinical information on patients, so that we can direct them to the right accompanying patient.’’ (E1-06-T2)

#### Ethical guidelines

In T1, APs mentioned having some concerns that some HPs would regard their interventions negatively out of concern that APs might discuss clinical details about treatments with patients. Initially, professionals were very reluctant to refer patients to us, as they were afraid we would give them false information. (E1-04-T1).

In T2, APs even went so far as to say that they were perceived as a burden that only “*would bring extra work*” (E4-03-T2) to the rest of the team, explaining HPs’ resistance to HPs’ integration. However, to some APs, this resistant attitude is less present than in T1:

"We really saw a change in attitude from the professionals over time, when they got to know us and saw that we were very aware of the limits of our interventions." (E4-03-T2).

"The reluctance of the doctors was overcome by the presentations, by the reassurance, on an ethical and legal level, of the legitimacy of the accompanying patients, and by the fact that we were introduced to the team." (E3-05-T2)

### Culture

#### Opportunities for discussion and dialogue

Related to the perception that the culture was not seen as being open in T1, APs had trouble meeting the managers to discuss implementation of the project and their interactions with patients. Once the project was implemented, APs in T2 did not have many opportunities to share with the rest of the team their feedback on the implementation of the project and roles with patients. In fact, during discussions with patients, APs gathered very useful information for improving the quality of care. Interactions with other HPs were limited: "*I wasn't sure who was who and who was in charge of the project*." (E1-03-T1).

However, the transmission of information between APs and HPs was done in T2, at 2 sites (E2 and E3), APs feel that “*an openness has been created*” (E2-01-T2) by HPs and “*they are very attentive*.” (E2-02-T2).

"When I had access to the patients, I thought it was important to be able to share the information gathered with the team when it seemed relevant, but this was not well organized. As we work mainly by telephone, it's important to have someone to help us keep in touch with the clinicians." (E1-05-T2)

To facilitate communication and dialogue, APs in T1 mentioned increasing opportunities for contact with HPs in order to receive verbal feedback that would help them adjust their work to their patients’ needs, and thus maximize therapeutic follow-up provided by HPs.

"Between us and with the support of the research team, we have built a logbook that we fill in after each interview with the patients. It's factual information, and if there's anything important that could change the medical course of action, we also report it verbally to the PA coordinator." (E3-04-T1)

#### Promoting the program

Some team members were well aware of APs’ support services, but did not necessarily always pass on the information to patients. One reason addressed both in T1 and T2 as to why HPs were not promoting APs to their patients may have been because they did not see it as a value-added: "*Professionals often forget to tell their patients that we exist; they haven't yet acquired the reflex to refer them to us*." (E1-08-T2).

An element mentioned in T2 by the APs was that they could be the one promoting the program to the clinical staff and the patients: “*Finally, the best thing would be for us to be the ones to promote the program to professionals and patients by being on the spot in the department*.” (E1-01-T2).

#### Recognition

APs felt as if the institution approved of the program and their inclusion in the team, but in practice, failed to give sufficient feedback to the APs to recognize their added value in certain establishments: "*There is a lack of places where we can find out how professionals appreciate our work*." (E1-07-T2).

In other cases, the creation of a community of practice bringing together all the APs enables them to exchange views with professionals and thus share their contributions to the team:

‘’During our Community of Practice meetings, we invite healthcare professionals to meet with us and discuss our work with them. This enables us to better perceive our added value in their work and observe how we can better meet their needs and those of their patients.’’ (E3-07-T2)

### Resources

#### Time

From T1 to T2, APs felt that the clinical staff had insufficient time to allocate to the program. This could have led to the clinical staff’s insufficient involvement in promoting APs’ services which, according to some APs, could have been partially responsible for the PAROLE-Onco program growing at such a slow pace. They added that, ironically, it is also this issue of time that set APs apart, emphasizing the importance of their presence within the clinical team: “*Professionals are overworked, and don't often think of referring their patients.*” (E1-04-T2).

#### Office space

In T1, APs mentioned that they did not have a dedicated space, such as an office, to be able to meet with their patients in a trust-inducing, discreet and confidential environment. Providing them with a peaceful and private area that is conducive to patients feeling comfortable to talk was thus mentioned as a potential solution. They also mentioned their need for an office to be able to promote the services offered and to enhance communication with the clinical staff. In an office, they could also discuss with other APs experiences they had had with their patients and share tips they had developed, for instance.

‘’We don't have a space where we can meet with patients in the facility, which poses a real problem in terms of being able to talk safely. It would be very useful if we could have an office where we could take time with the patient and do it in a place where there's no risk of being overheard.’’ (E3-02-T1)

Two establishments subsequently created offices to accommodate the APs: "*In the centre that has been created we have our own space; we have an office where we can chat with patients*." (E3-06-T1).

#### Financial compensation

In T1, not having financial compensation for their work was discussed as having limiting impacts on their motivation to persevere in the program while also reinforcing the idea of lack of recognition they felt a need for financial support, to cover both travel and parking, for instance, in order to declare their legitimate presence within the clinical team. Some suggested that they could be provided with a budget, which would compensate them for the cost of the parking ticket, mileage charges or lunch at the cafeteria for instance.

‘’Personally, I'm not in it for the money. Then, I don't think it would be a means, unless you had an extraordinary volume, you know it wouldn't come in the first place, it wouldn't be a facilitating means. You're not doing this for money. You're really doing this to invest yourself, and then try to give as much as you can [...].’’ (E1-03-T1)

However, in T2, even if these measures were implemented, APs did not consider financial compensation an essential factor in their involvement in the program, as accompanying patients fulfilled their desire to help others and give back. Nonetheless, some APs suggested that the volunteer aspect of the program be maintained for some, and that other APs, who would be taking on more demanding tasks such as physically accompanying patients to their appointments, be remunerated. Others proposed waiting until the program reached a larger scale with a number of volunteers substantial enough to justify implementing a form of salary.

‘’The matter of remuneration is complex. It can't be summed up as a yes or no answer. It depends on the time spent, the activities carried out, the status of the APs, who may need money to be able to invest, or on the contrary, if they are paid, they may not be able to benefit from other financial support.’’ (E2-02-T2)

### Tools

#### Community of practice

In T1, APs mentioned that having a community of practice could be beneficial for them to improve their work. In T2, APs have seen the benefits of having this space of sharing for the community, although the community of practice only exists in 2 establishments. *“I love taking part in the community of practice, we all share our experiences together and I learn a lot from my colleagues.”* (E1-07-T2).

Thus, some APs suggested implementing meetings at a provincial level from different establishments to be able to share their knowledge, answer each other’s questions and recruit more APs. They believe that if they “*could feed off of, then equip [them]selves with the experiences of other sites, it would be greatly beneficial*." (E1-01-T2).

#### Notes in patients’ files

Another tool that would be useful to APs is the inclusion of notes in the patients’ medical files for HPs to consult. However, only one establishment has implemented this system (E2) on a regular basis. Another way of transmitting information on patients to HPs is to maintain logbooks which are intended for research purposes but could also be used in the clinic. Some APs use them to relay information to the team.

‘’From the outset, we obtained from the establishment the possibility of having a sheet in the patient's file dedicated to our interventions. This enables us to make a summary of our consultations with patients that professionals can consult.’’ (E2-01-T2)

‘’After each intervention, we fill in the logbook, which is shared with the AP coordinator.’’ (E3-03-T2)

#### Phone meetings

In T1, APs mentioned that due to the COVID-19 pandemic distancing measures, accompanying sessions were done by phone, which was more effective than what had initially been anticipated in T1. These telephone meetings were less emotionally charged for APs as the remote nature helped them distance themselves from their patients’ words and, as a result, a more objective environment could be fostered. Telephone meetings also provided an intimate environment of communication and discussion that encouraged patients to open up.

‘’Initially, I began my accompanying work in the presence of patients. Then with the pandemic, we no longer had access to patients. So we started doing it over the phone. I wasn't in favour of it at first, but then I realized that it was easier for patients to open up and share their emotions.’’ (E2-01-T1)

## Discussion and recommendations

The purpose of this study was to better understand, from the perspective of APs themselves, the uncertainty and ways to improve their integration into clinical oncology teams, which are two key elements while implementing complex intervention [26]. Although there are areas of continuity between T1 and T2 in the identification of limiting and facilitating factors, APs offer a more concrete evaluation of the needs in T2. For example, in T1, APs addressed organizational heaviness and a lack of energy to implement PAROLE-Onco as the main limiting factor. Conversely, in T2, they talked about solutions, such as having a resource person and meeting directly with the team members to convince them and reduce the reluctance and lack of understanding about their activities. A satisfaction questionnaire was also suggested to be conducted to collect comments and improve their practices. This change in vision may come from the experience of APs who, over time, were able to better analyze and identify the best factors for successful implementation. Also, APs in T2 encountered less resistance than in T1. This can be seen as a testament to the change in culture that has taken place since the beginning of the project to not only implement, but now to sustain the project, particularly in three establishments.

To better interpret the results, compare with the literature and draw lessons for the implementation of complex interventions, we mobilized the Consolidated Framework for Implementation Research (CFIR) [21, 22].

### Intervention characteristics

The first characteristic of this intervention was that neither the people who were going to make it (the APs) nor those who were going to receive it (decision-makers, managers, and HPs) knew how it was going to be carried out. This created a great deal of uncertainty among the various players involved. The very nature of innovation, of having to deal with a blank page, leads to mechanisms of resistance to change. What's more, the intervention is part of the relatively young care and service partnership movement, which recognizes patients' expertise in relation to their experiential knowledge of living with illness and using the healthcare system [27]. This philosophy of care is not yet fully implemented in Quebec [28] or elsewhere in Canada [29] and the world [30], so professionals are being challenged in their relational schema with patients. Finally, the intervention was implemented both face-to-face and by telephone, and in both cases, the strengths and weaknesses were noted by the APs. These different modalities also exist in the literature [31].

### Implementation process

Referring to the activities and strategies used to implement the innovation, this domain includes four constructs: planning, engaging, executing and evaluating. To achieve this, the success factors that emerge from our study focus on the need for a project manager who has a fairly clear idea of the project and is able to anticipate all stages of program implementation. This requires strategic and operational governance committees to be created, and to include accompanying patients from the outset, so that decisions take into account patients’ points of view [14]. Implementation also requires the constant involvement of APs to ensure that they can adjust their involvement and support deployment, as well as making professionals aware of the importance of their commitment to its implementation and maintenance over time [21, 22]. At last, having a program evaluation with patient- and system-level outcomes could be instrumental in leadership support and in improving the quality-of-service provided [13, 32]. The evaluation results could be essential to showing the impact internally and to supporting the program’s growth in terms of being better aligned with patient needs [14, 16].

### Characteristics of individuals

This domain corresponds to the roles and characteristics of individuals involved in implementation, whether it is the knowledge and beliefs, the individual stages of change, the leadership, and those delivering innovation. The knowledge and beliefs of HCPs and managers on APs are very weak. No one had the opportunity before to work with patients as partners to introduce and innovation and to work as colleague. Not knowing what an AP could do, not knowing how to work with them, the results illustrate that professionals and managers have a great deal of room to learn, and this lack of knowledge leads to resistance to implementing the changes needed to introduce APs into the team. The results also show the importance of shared leadership at all levels of project governance, from the CEO through to the project manager, managers, clinicians and patients themselves [21, 22]. The importance of identifying the champions or early adopters promotes implementation. Finally, the innovation deliverers, aka the APs, also have very specific characteristics. They are people who want to give meaning to their cancer journey, to give back to the next person and to provide the support they have missed during their journey [11]. They are also people who are not used to having this kind of relationship with professionals, they have to prove themselves and show their added value to the team. In addition, for now, the recognition of APs’ status in the healthcare establishments is not clear and can lead to confusion [33].

### Inner settings

This domain corresponds of the healthcare establishments’ characteristics in which the APs are implemented [21, 22]. Retained constructs include relational networks and communications, culture, structural characteristics and available resources and materials solicited during the implementation process.

In terms of formal and informal networks and communication, it has been found that facilitators included robust administrative support, program functioning and team cohesion [13, 15]. Conversely, developments in mental health peer support programs highlighted the fact that implementation in a healthcare setting can be hampered by role confusion, inadequate training of HPs working with peers or lack of professional development, and the slow pace of development [13, 14, 27–30, 32, 33]. This is consistent with some of our results in terms of governance, where defining the boundaries of APs’ intervention initially slowed down the decision-making process essential to program growth [32, 34]. HCs may fear that APs will provide incorrect information and make their work more complex. Although APs’ integration was slow at first, it allowed them to take the time to reassure team members that their role as APs and their relationship with patients were valuable and safe. Therefore, harmonizing APs’ roles with the rest of the team could help prevent role confusion and increase APs’ recognition, leading to their contribution making a higher impact [35]. To this end, APs suggested teaching HPs how APs’ activities are complementary to theirs and how they could work with APs. Training for professionals and patients on how to work together helps integrate peers into clinical teams [36]. In addition, at the beginning of the project, APs cited communication challenges with the clinical team and managers. They raised few opportunities to discuss the program’s implementation and continuous improvement even if communication is key in helping to successfully implement the peer support program [13–15, 35]. In addition, communication between APs is a key factor in program implementation. Different peer support programs received support through multiple media platforms [32] or developed structures to support cross-institutional networking which allowed APs to communicate across different clinical sites [15]. Similarly, APs mentioned that having a community of practice inter-establishments creates a space to share tips and tricks with fellow APs with the objective of bettering their practice.

Culture refers to the sharing of values, beliefs, and norms centered around caring, supporting and addressing the needs and welfare of patients and APs [21, 22]. The identification of patients' needs by professionals to be emotionally supported by HPs does not stand out in the results. Also, the lack of perception of this need is detrimental to the mobilization of HPs [13, 32]. As a result, APs suggested that opportunities be created for team members to meet APs to share their expertise and how they interact with patients.

Regarding structural characteristics, APs raised the fact that the main facilitating factor was to have a resource person responsible for facilitating interactions between APs and the clinical team. This person also helps make the program sustainable [16].

Resources can cover different issues. For example, APs identified that staff members have a substantial workload and therefore have inadequate time to allocate to the program [14, 33]. However, entrusting APs with a certain task-sharing role could help alleviate team members’ workload. Having a clinical office space is described as being instrumental in the success of APs’ integration [12, 13, 35]. It enables APs to meet with patients and team members and explain the program to them, meet with other APs to share experiences, and coordinate the program by assigning patients to other APs. In our case, having a space was mentioned as being a symbol of recognition as highlighted by Huntingdon [37]. Similarly, having financial compensation was cited as an incentive for persevering in the program and being valued, even if it is not considered a key factor in APs’ involvement in the program. Finally, APs’ notes added automatically to the patients’ medical files or by maintaining logbooks help APs integrate more easily into the clinical team [35].

### Outer setting

**Fig. 1:**
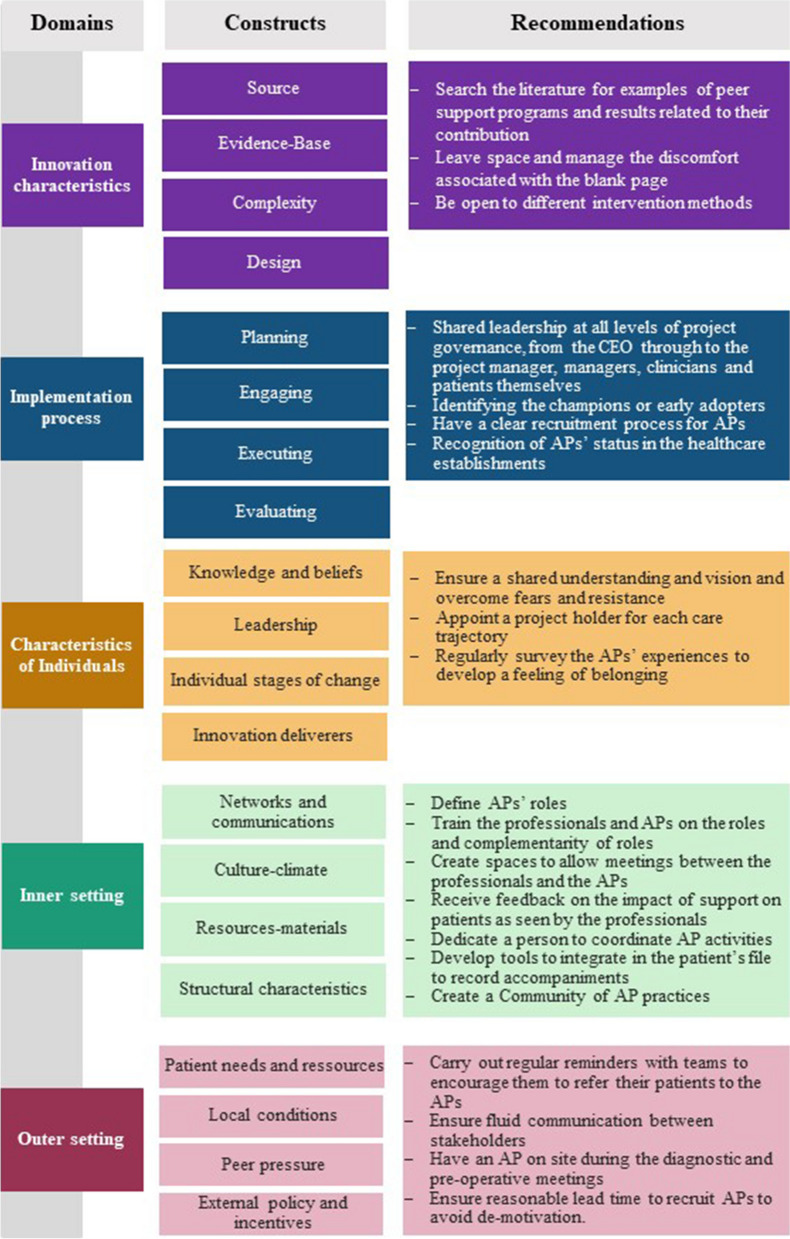
Factors leading to recommendations

The outer setting domain covers partnerships and connections, policies, financing and external pressure. The results of this study showed little evidence of partnerships between the 4 facilities. However, the development of a patients' medical files for HPs to consult influenced the other sites to create a logbook. These inter-facility exchanges were made possible by regular meetings between the sites, so that they could discuss their practices [38]. Policies in place in Quebec encourage care partnerships at facility level, but especially at organizational level. When the project was first introduced, no policy referred to such support. However, since then, the Ministère de la santé et des services sociaux has included the deployment of APs in oncology departments in its strategic axes [39]. In addition, a request for funding from the APs coordinator is underway to maintain this intervention over time. Figure 1 presents all the factors that led to the recommendations.

### Strengths and limitations

The concept of APs as an integral member of a clinical team is quite recent, especially because those navigators are former patients. Our study is exploratory and requires further study over time in addition to carrying out quantitative and qualitative studies to test different models. One of the strengths of this study is precisely that it examines implementation through the eyes of those most affected (APs) who are rarely asked for their perception on the implementation process. This analysis could be complemented in the future by a view from professionals and managers who may have a different perspective on the barriers and facilitators to implementation. We also recognize that APs have different perceptions of their integration, and therefore the results may not be an exact representation for all APs. In addition, based on the perception of APs, the proposed factors may be influenced by their personal experience as patients. Adding non-participant observation in accompanying sessions for research purposes and conducting a cross-analysis with HPs’ and patients’ perceptions could help to better assess the limiting and facilitating factors of APs’ integration into clinical teams. In addition, the contexts in the 4 establishments differ and, accordingly, our results cannot be generalized. It is also important to recognize that the anticipated facilitators and barriers discussed in this study could differ if this intervention were to be fully implemented, as this constituted the first phase of implementation of the PAROLE-Onco program. Moreover, here we have presented APs’ perspective of the challenges and facilitators of their integration, but it is also important to assess what their roles are and what the effects of those role are on themselves, on the patients and on the clinical team. Those results are presented in another manuscript [11]. Also, of the 29 APs that were included in the clinical teams at the 4 establishments, 20 participated in the study because some had changed positions or were unable to respond to our request. However, in our data collection process, both at T1 and T2, we felt that we had reached data saturation.

## Conclusion

The PAROLE-Onco program aims to evaluate the integration of APs as full-fledged members of the clinical oncology teams in a real context. This article focuses on the results of individual interviews with APs involved in the implementation to assess their perception of the factors influencing implementation of this program in 4 healthcare establishments. Our results show that misunderstandings on the part of HPs about APs’ roles can explain the delayed change in culture in healthcare establishments and therefore increase the difficulty experienced in promoting accompanying services by APs. Creating opportunities for the clinical team members to exchange information with APs, build trust and recognize APs’ value in the team are identified as key facilitating factors. Furthermore, creating spaces by and for APs for sharing tips is essential to developing a community that strives to better their practice. Therefore, giving certain powers to APs, through co-construction and co-decision methods, is favourable to achieve a change in culture within healthcare establishments. This study highlights the importance of working proactively during the implementation with APs and HPs on organization, leadership, resources and status factors to allow patients dealing with cancer to benefit from the experiential knowledge of other patients within their clinical team.

### Supplementary Information

**Additional file 1.**

## Data Availability

All data generated or analysed during this study are included in this published article [and its Supplementary material file].

## Abbreviations

AP: Accompanying patient
CHU: Centre Hospitalier Universitaire
CHUM: Centre Hospitalier Universitaire de Montréal
CISSS: Centre Intégré de Santé et de Services Sociaux
CIUSSS: Centre Intégré Universitaire de Santé et de Services Sociaux
COVID-19: Coronavirus Disease 2019
CR-CHUM: Centre de recherche du Centre Hospitalier de l’Université de Montréal
PAROLE-Onco: Patient Advisor, an Organizational resource as a Lever for an Enhanced Oncology patient experience
HPs: Healthcare professionals
E1-E4: Establishments
E1: Centre hospitalier de l'Université de Montréal
E2: Centre Hospitalier Universitaire de Québec-Université Laval
E3: Centre intégré universitaire de santé et de services sociaux (CIUSSS) de l’Est-de-l’Île-de-Montréal
E4: CIUSSS de la Mauricie-et-du-Centre-du-Québec
T1: First time of evaluation, beginning of APs intervention
T2: Second evaluation time, two years afterwards
QDA Software: Qualitative data analysis software
CEO: Chief Executive Officer
CIFR: Consolidated Framework for Implementation Research
